# Cognitive Relapse in Multiple Sclerosis: New Findings and Directions for Future Research

**DOI:** 10.3390/neurosci3030036

**Published:** 2022-08-26

**Authors:** Zachary L. Weinstock, Ralph H. B. Benedict

**Affiliations:** 1 Buffalo Neuroimaging Analysis Center, Department of Neurology, Jacobs School of Medicine and Biomedical Sciences, University at Buffalo—State University of New York, Buffalo, NY 14203, USA; 2 Jacobs MS Center for Treatment and Research, Department of Neurology, Jacobs School of Medicine and Biomedical Sciences, University at Buffalo—State University of New York, Buffalo, NY 14203, USA

**Keywords:** multiple sclerosis, cognition, relapse, cognitive relapse, neuropsychology, SDMT, memory, MRI, cognitive impairment, cognitive rehabilitation

## Abstract

Multiple sclerosis (MS) is a chronic inflammatory, demyelinating disease of the central nervous system, often presenting with brain atrophy and cognitive impairment (CI). In the relapsing–remitting phenotype, cognitive performance is increasingly recognized to decline acutely during MS relapse, with varying degrees of recovery afterwards. Therefore, CI in MS may result from incomplete recovery from episodes of so-called “cognitive relapse”, gradual neurodegeneration, or both. Among a variety of validated measures of cognitive performance, the Symbol Digit Modalities Test (SDMT) represents the most sensitive measure of cognitive decline and is easily translated to clinical practice. In fact, cognitive relapse identified using the SDMT has been reported in clinically relapsing cohorts as well as in individuals with no other neurological signs, suggesting that routine cognitive assessment may be necessary to fully appreciate the extent of a patient’s disease activity. The aim of this narrative review is as follows: (1) to provide the historical context for neuropsychological assessment in MS, (2) to provide a summation of key studies describing the cognitive relapse phenomenon, and (3) to discuss current gaps in our knowledge and highlight avenues for future research.

## 1. Introduction

Multiple sclerosis (MS) is an inflammatory, demyelinating, and neurodegenerative disease of the central nervous system (CNS) often presenting with cognitive impairment (CI) [1]. Although the clinical course of MS varies considerably between patients, the vast majority (~83%) of people with MS (PwMS) are diagnosed with relapsing–remitting MS (RRMS), which is characterized by episodes of acute neurological symptoms followed by remission. Later, RRMS transitions to a secondary-progressive course (SPMS) marked by incomplete recoveries following relapse, as well as a steady progression of neurological disability independent of relapse activity [2]. The CI in PwMS may stem from incomplete recovery after relapse, gradual neurodegeneration, or both. The former process, dubbed “cognitive relapse” has only recently been described in the literature [3,4,5,6]. Here, we aim to provide a narrative review of cognitive relapse in MS with a summation of key studies, and a discussion of how gaps in our knowledge may be addressed in future work. By way of background, we begin with a brief introduction of CI in MS.

## 2. Historical Review of Cognition and Neuropsychological Assessment in MS

Despite Jean-Martin Charcot’s recognition of “a marked enfeeblement of the memory” and “conceptions that are formed slowly” in the late 19th century [7], little was known about cognitive function in MS until psychometric testing was applied beginning in the 1980s. A series of large-scale, controlled studies using batteries of standardized neuropsychological measures showed that CI in MS was far more common than previously thought [8,9]. Taken as whole, these studies demonstrated that CI impacts closer to 45–65% of MS patients, is more common in SPMS than RRMS, is only weakly correlated with physical disability, and is sorely underrecognized in the absence of objective neuropsychological assessment [10,11].

In the years following their introduction to MS research, neuropsychological testing protocols have markedly evolved, trending towards shorter batteries that are more easily administered in a clinical setting. For example, in 1990, Peyser et al. proposed a 2 h “core” battery of 17 cognitive tests that was considerably more feasible than the up to 6 h needed for a comprehensive neuropsychological assessment [12]. Shortly thereafter, a seminal study by Rao demonstrated that, amidst a similar battery of 23 tests, a subset of 4 indices discriminated cognitively intact and impaired MS patients with a sensitivity and specificity comparable to the rest of the tests combined [11]. Adapting these four tests into the Brief Repeatable Battery (BRB), Rao formed the first standardized cognitive screening tool for PwMS [13]. In 2002, an expert panel developed the Minimal Assessment of Cognitive Function in MS (MACFIMS) which included similar tests with superior psychometric properties, as well as additional measures of executive function and spatial reasoning [14]. However, despite comparing favorably to the BRB, the additional tests in MACFIMS contribute to a testing time of 90 min which, while initially regarded as brief, came to be known as too lengthy [15]. Therefore, most recently, an international expert panel recommended a set of three tests, which they named the Brief International Cognitive Assessment for MS (BICAMS) [16]. Taking just 15 min to administer, the BICAMS screens for deficits in processing speed, verbal memory, and visuospatial memory, using the Symbol Digit Modalities Test (SDMT) [17], California Verbal Learning Test–II (CVLT-II) [18], and the Brief Visuospatial Memory Test-Revised (BVMT-R) [19], respectively. The measured cognitive domains are considered to be three of the most commonly impacted in MS, and the chosen tests are now widely regarded as the most sensitive tasks available for cognitive monitoring in MS [20]. Among these, the SDMT stands out as the most sensitive, and it has garnered recognition as the gold standard assessment for cognition in MS [21]. Indeed, the SDMT is the sole test common to all three of the major cognitive batteries (Table 1), and is now recommended as an endpoint in clinical trials [21,22]. Further, work is underway to incorporate the SDMT into the Expanded Disability Status Scale (EDSS), the most common measure of disability in MS [23,24].

## 3. The Identification of “Cognitive Relapse” in MS

Prior to 2011, a handful of case reports and observational studies suggested that acute cognitive changes might occur during or after relapse [30,31,32]. However, these studies were limited, as they either failed to control for baseline cognitive status or lacked a control group. Serendipitously, data from the Safety of Tysabri Re-dosing and Treatment (STRATA) study would empower Morrow and colleagues to address these issues [33]. 

The STRATA study was designed to extend the safety profile of natalizumab by tracking a cohort of patients receiving natalizumab over the course of a year. A chief concern was the potential development of progressive multifocal leukoencephalopathy (PML). Therefore, prior to each monthly infusion, participants were screened for cognitive changes that might indicate its onset. Among these screening measures were the SDMT and the MS Neuropsychological Screening Questionnaire (MSNQ) [34], which screens for self-reported cognitive difficulties. Although no cases of PML were observed, 53 cases of confirmed MS relapse were identified during the study. Serving as “cases”, their raw SDMT scores from before, during, and after relapse were compared with 115 clinically stable “controls” who were matched on the basis of age, sex, baseline SDMT score, and time from study initiation. Notably, the authors discovered that the change in SDMT score between the visits immediately preceding and following an identified relapse was significantly different between cases (−1.2) and controls (+1.3). Furthermore, SDMT performance among cases was observed to recover following relapse, lending additional support to the transient nature of the observed cognitive change. Therefore, for the first time, Morrow and colleagues provided convincing evidence that cognition may be impaired by MS relapse.

While the work led by Morrow was a crucial step towards the recognition of cognitive dysfunction during MS relapse, it also had a number of limitations, largely owing to its retrospective nature [33]. First, the monthly assessments were not designed to coincide with relapse onset. Further, all participants analyzed were treated with natalizumab and it is not yet understood whether and how natalizumab interacts with relapse severity, especially with respect to its cognitive component. Lastly, other neurological variables that could influence cognitive performance were not measured; the SDMT is a visual test and optic neuritis—acute inflammation of the optic nerve affecting vision—is an obvious confounding factor. 

In 2014, Benedict et al. endeavored to study acutely relapsing MS patients with evidence of cognitive worsening by self-report, informant report, or clinician impression [5]. Crucially, the authors utilized a prospective design, evaluating participants’ cognitive status at a clinically stable baseline, again on the day a relapse was identified, and a third time at a 3-month follow-up. Furthermore, patients with optic neuritis or spinal cord signs which might confound cognitive testing were excluded. In contrast to a sample of matched clinically stable MS participants serving as a control group, only the relapsing group evidenced significant changes to SDMT performance between study timepoints. Specifically, the relapsing group declined by 3–4 points on the SDMT at relapse followed by a recovery of the same magnitude at the 3-month follow-up, whereas the stable group trended towards improvement across all timepoints, likely owing to a practice effect. Therefore, for the first time, cognitive functioning was observed to vary directly with MS relapse status through the use of a well-validated neuropsychological testing instrument in a prospective study design. 

**Figure 1:**
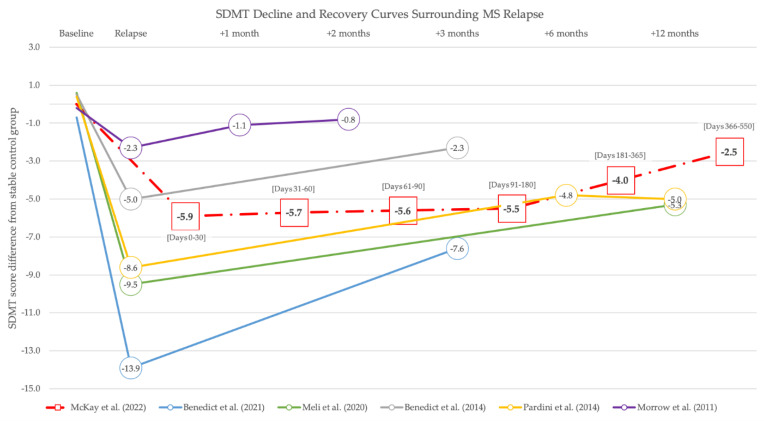
The SDMT decline and recovery curves surrounding MS relapse. The difference in raw SDMT scores between relapsing and stable MS patients across a variety of patient populations and timescales is shown. For each study, the mean score of the stable control group is subtracted from the mean score of the relapsing group. McKay et al. (bold text, red dashed line) represents the latest and most comprehensive study to date of cognitive change around MS relapse [35]. Because McKay used a national clinical database rather than a controlled study with discrete timepoints, each datapoint represents aggregate data from one of six distinct periods following relapse onset, indicated in brackets as the number of days post-relapse. Relapsing and stable groups were well matched at baseline across all studies with difference scores ranging from −0.7 to 0.6. Included for reference are data from studies of cognitive relapse described elsewhere in this paper such as those led by Benedict [5,36], Meli [3], Pardini [6], and Morrow [33]. Cognitive recovery after relapse exhibits marked variation across studies but is generally considered incomplete.

In the years following Morrow and Benedict’s recognition of relapse-associated cognitive decline, the phenomenon of cognitive relapse was reported by other research groups (Figure 1) [3,4,35]. However, most of these studies followed subjects for only 3 months after relapse and, therefore, the long-term impact of cognitive relapse on cognition remains unclear. 

Pardini and colleagues reported in 2014 that clinically meaningful declines in SDMT (defined as a decrease of at least 4 points) occur in the absence of other clinical signs [6]. The authors monitored SDMT, MRI, and neurological status at 6-month intervals. Selecting those with a decline in SDMT, it was noted that the drop in performance was associated with gadolinium enhancing lesions on an MRI, but not a change in EDSS. Moreover, observed recovery was not complete, again suggesting that some patients do not recover and that this may account for some of the variance in progressive cognitive decline. It is now debated if “isolated cognitive relapse” is a true phenomenon, awaiting replication.

Recently, a landmark study by McKay and colleagues attempted to address the dearth of long-term cognitive recovery data by harnessing the considerable power of the Swedish MS Registry (SMSreg), a national repository of clinical and cognitive data prospectively collected from MS patients in Sweden [35]. Focusing on patients with relapsing–remitting MS, active disease, at least 2 SDMT scores, and a minimum of 3 years of follow-up, a cohort of 3877 cases was defined. Among these patients, more than 31,000 distinct SDMT scores were collected across a mean follow-up of 10.7 years. Notably, after controlling for age, sex, practice effect, and disease-modifying therapy status, the authors demonstrated that, relative to periods of clinical stability (i.e., “remission”), SDMT performance significantly declined as early as 30 days pre-relapse. This finding suggests that acute worsening of cognitive processing speed as measured by the SDMT may reveal subclinical brain inflammation and herald an impending clinical relapse. Furthermore, the observed effect of relapse on SDMT lasted substantially longer than previously reported, as participants’ performance only returned to the level of remission after 18 months had elapsed. Last, the scale of this study in terms of sample size and length of follow-up should remove any doubt that cognitive relapse is an important facet of active MS.

## 4. The Evolving Understanding of Cognitive Relapse

While the work of Morrow, Benedict, and McKay was integral to the establishment of cognitive relapse as a now widely recognized phenomenon in MS, there remain considerable gaps in our knowledge. For example, whether and how cognitive domains other than processing speed are impacted during MS relapse remains unknown. Additionally, as shown in Figure 1, the magnitude of cognitive change before and after relapse varies considerably between populations and individuals, raising questions as to which factors might confer susceptibility to cognitive decline, resilience or reserve influences on recovery, and likelihood for future progression of CI. Moreover, the existence of cognitive relapse offers a new dimension by which MS disease activity might be assessed [37]. In the third and final part of this review, we endeavor to address recent efforts to better understand the cognitive dimension of MS relapse, and highlight avenues of future research.

Currently, the available literature regarding cognitive relapse focuses overwhelmingly on cognitive processing speed as measured by the SDMT. This is not surprising, as the SDMT is the only neuropsychological test to consistently reveal significant decline during MS relapse [1]. However, as recently highlighted by Leavitt, to focus solely on the incredible sensitivity of the SDMT is to neglect the specificity offered by other measures [38]. This in turn, limits a broader characterization of the various cognitive deficits which might arise during MS relapse. For example, in a recent report examining cognitive recovery after relapse, Benedict and colleagues described a low-moderate effect (d = 0.4) and trend towards significance for the group x time interaction of the CVLT-II in a cohort of relapsing and stable MS participants [36]. Similarly, although lacking baseline cognitive data, Giedraitiene observed significant improvements after relapse on both the BVMT-R and CVLT-II [4]. Therefore, future research should continue to employ alternative neuropsychological tests to more richly characterize cognitive relapse and determine the frequency with which different cognitive domains are impaired. 

As noted previously, the extent of decline on the SDMT varies widely between studies with relapsing and stable MS participants separated at relapse by as few as 2.3 raw score points [33] and as many as 13.9 raw score points [6]. This raises an obvious question—what factors contribute to the severity of relapse-associated decline? Past MRI studies have shown moderate correlations of acute MS pathology, such as contrast-enhancing white matter (WM) lesions, with cognitive status [39,40,41]. Furthermore, acute inflammatory activity may interact with chronic MS pathologies, such as non-enhancing WM lesions and volumetric brain changes, as well as with potentially protective factors, such as cognitive and brain reserve [42]. 

Equally important is the extent of cognitive recovery which follows cognitive relapse. Some studies have demonstrated a return to control-level performance within 2–3 months [4,33], whereas McKay and colleagues reported maintenance of clinically meaningful declines on SDMT at up to 18 months post-relapse [35]. This substantial variability in post-relapse outcomes underscores a key concern of both patients and physicians—there is currently no way of anticipating which patients will be able to return to their usual way of life in the wake of a relapse. Benedict and colleagues recently reported that among various clinical, demographic, and MRI measures, only participants’ level of education—a common proxy of cognitive reserve—predicted SDMT recovery after relapse. A promising alternative was recently presented by Weinstock et al. wherein the authors demonstrated that thalamic volume during relapse predicted SDMT recovery at a 3 month follow-up [43]. Although this requires a degree of additional MRI processing beyond standard radiological interpretation, it represents at least one method by which an individual’s “reserve” against further MS pathology might be quantified in order to guide clinical decision making.

So-called isolated cognitive relapses (ICRs) are reported [3,6] but replication is needed. By the standards originally put forth by Pardini et al., ICRs are defined as follows: (1) by a clinically meaningful decline of at least 4 points on the SDMT, (2) by the presence of gadolinium-enhancement on MRI indicative of acute disease activity, and (3) by the absence of clinical or subjective evidence of new neurological signs or symptoms. Using these criteria, 17 cases of ICR were retrospectively identified among a cohort of 99 subjects [6]. Notably, despite matching the cognitively stable sub-cohort on the basis of baseline SDMT performance and cognitive reserve variables, the ICR group maintained significantly poorer performance at both 6- and 12-month follow-up visits, mirroring the incomplete recovery observed in most studies of cognitive relapse [1]. Interestingly, however, the ICR group did not significantly decline on a self-reported version of the MS Neuropsychological Screening Questionnaire (MSNQ) which screens for cognitive difficulties [34]. This finding has since been used by critics to question the purported existence of ICR [44]. It is worth noting, however, that in PwMS, self-reports of cognitive performance are more strongly correlated with depression than cognition, whereas the opposite pattern is observed when using informant reports [45]. Indeed, a subsequent study of ICR by the same research group reproduced Pardini’s original findings and also demonstrated that ICR presents in association with significantly increased cognitive difficulties, as evaluated by an informant-report version of the MSNQ [3]. In summary, although the existence of ICR is (and perhaps should be) viewed with at least a degree of skepticism, it may represent a fundamental shift in what is considered disease activity in MS. 

What is the neural basis of cognitive relapse? There are some data suggesting the number or volume of gadolinium enhancing lesions is, as would be expected, a significant factor [5,6]. There are, however, other molecular or inflammatory factors that might contribute to relapse-associated cognitive decline. For instance, inflammation is understood to alter synaptic function, likely contributing to brain network dysfunction [46]. As such, recent work by Trenova et al. revealed significantly more pro-inflammatory cytokines in cognitively impaired MS as compared to their cognitively preserved peers [47]. This explanation was further supported by McKay et al. wherein cognitive decline was observed up to 30 days prior to the clinical recognition of relapse, an observation the authors attributed to subclinical inflammation [35]. This study was limited, however, by a lack of neuroimaging data and, therefore, the authors could not account for structural brain changes that may have otherwise been visible on an MRI scan.

The clearest explanation focuses on the gadolinium-enhancing lesions seen on MRI scans, which serve as surrogate markers of inflammatory activity. Several studies have now revealed an association of gadolinium enhancement on MRI scans with cognitive dysfunction, even in the absence of a clinically-defined relapse [39,40,41]. However, the mechanism by which gadolinium-enhancing lesions impact cognition remains unclear. One theory implicates a direct effect of the disruption of WM streamlines connecting specific brain networks [48]. Indeed, in their investigation of ICR, Pardini and colleagues noted patterns of gadolinium-enhancing lesions between the ICR and non-ICR groups [6]. It is worth noting, however, that the impact of such lesions on specific structural brain networks has not yet been assessed in a prospective study of cognitive relapse. Meanwhile, an alternative hypothesis posits that the blood–brain barrier breakdown responsible for expression of gadolinium enhancement contributes to a much broader infiltration of the brain parenchyma by the immune system, resulting in diffuse synaptic dysfunction as described previously [49]. Further study is required to determine whether and to what degree gadolinium-enhancing lesions and molecular inflammatory markers provide overlapping vs. independent explanatory value.

A further noteworthy candidate that might influence cognitive changes around MS relapse is that of reserve. Recently, a study by Benedict and colleagues demonstrated that education, a proxy of cognitive reserve, significantly contributed to the prediction of cognitive recovery after relapse [36]. Although cognitive reserve is most commonly conceptualized as a gradually adaptive process [50], it remains to be seen whether and how it might interact with acute cognitive change during MS relapse. Relatedly, a role for brain reserve in cognitive relapse was supported by work recently presented by our group, in which normalized thalamic volume at the time of relapse predicted cognitive recovery on the SDMT at a 3 month follow-up [43]. Together, these data demonstrate that further study of reserve variables is indicated to clarify the roles of both cognitive and brain reserve in the context of cognitive relapse.

Worth additional consideration are psychological comorbidities, such as depression and anxiety, which are more common in PwMS as compared to the general population [51]. Such factors are widely acknowledged to influence cognitive performance, confounding the interpretation of neuropsychological test results. Put simply, these factors might influence cognitive change during relapse as opposed to new pathology/active disease. Unfortunately, in the context of cognitive relapse, few studies have looked at potential associations with psychological factors, and the data that are available are mixed. For example, Weinstock et al. found no difference between cognitively relapsing and stable MS patients on a self-report survey of depression [52]. This stands in contrast to a recent study which suggested that anxiety and depressive disorders in MS are associated with dissociable patterns of cognitive impairment, although participants’ disease status (e.g., “relapsing” vs. “stable”) was not reported [53]. Therefore, further research is indicated to determine whether these factors influence a patient’s susceptibility to cognitive change during relapse or perhaps, the degree of decline or recovery surrounding cognitive relapse. 

Were these factors to influence cognitive change around relapse, investigators ought consider whether mindfulness-based approaches from the “third-wave” of cognitive behavioral therapy (CBT) might aid patients’ recovery after relapse [54]. A recent review of the available literature suggested that such therapies—which emphasize concepts such as acceptance and compassion—may be capable of treating a range of psychological difficulties in the MS population, including depression and anxiety [55]. Accordingly, third-wave CBT approaches might indirectly improve cognitive functioning after relapse via their impact on a patient’s psychological status. In support of this notion, a 2010 review of mindfulness training in generally healthy cohorts revealed the positive effects of such interventions on cognitive functioning [56]. It remains to be seen, however, whether such therapies are beneficial in the post-acute or early stages of relapse recovery in PwMS. 

As alluded to previously, the potential implications of cognitive relapse are profound. Currently, unlike motor or sensory changes, cognitive change alone is not considered sufficient evidence of disease activity in MS. While studies of ICR suggest this to be a mistake, it is likely a result of the relative paucity of data on ICR, as well as the inertia of clinical practice. Nonetheless, emerging research continues to support the inclusion of cognitive change as a standard clinical sign of disease activity. For example, among a cohort of clinically stable MS patients who achieved no evidence of disease activity (NEDA) status [57], nearly 60% of participants demonstrated longitudinal worsening in two or more cognitive domains [58]. It is therefore clear that a failure to recognize longitudinal cognitive change in MS is a failure to recognize disease progression which could in turn delay the offer of rehabilitative therapies [1]. In support of this notion, a consensus group recently recommended the clinical capture of SDMT performance soon after diagnosis to serve as a baseline against which future cognitive performance may be measured [59]. Data, such as these, might then be coupled with evidence-based recommendations, such as in a recent work by our research group which proposes a threshold marking statistically reliable decline on the SDMT [52]. If incorporated into clinical routine, this could empower clinicians to better screen for evidence of cognitive relapse and, more generally, MS disease activity. In fact, efforts are already underway to incorporate objective tests of cognition into the EDSS, the most common clinical measure of disability in MS [24]. A further recommendation advocating for the inclusion of the SDMT as an outcome measure in clinical trials may also expand our understanding of the cognitive impact of disease-modifying therapies in MS [21]. In summary, the translation of neuropsychological assessment to routine clinical practice is absolutely necessary if we are to better recognize disease activity, track disease progression, and judge drug efficacy.

Beyond the immediate clinical implications involving cognitive change as a marker of disease activity, much remains to be learned about the neuropathological changes driving cognitive relapse, as well as the impact of such relapse on functional trajectories. As discussed previously, the factors influencing acute cognitive decline during relapse, as well as cognitive recovery after relapse, remain poorly characterized. For example, whether cognitive change during relapse is a direct result of WM/brain network disruption, an inflammatory cascade, or some combination of the two, and how these markers of disease activity interact with cognitive and brain reserve remains unknown. Moreover, whether and to what degree longitudinal cognitive decline in PwMS is a reflection of the neurodegeneration expected of otherwise clinically stable patients as opposed to an incomplete recovery following cognitive relapse is unclear. In brief, there are substantial gaps in our understanding surrounding relapse-associated cognitive decline that must be filled if we are to better understand and address the cognitive dimension of MS relapse.

When involving cognitive impairment, MS relapse is reminiscent of the acute cognitive declines found in mild traumatic brain injury and concussion. In both, there is an acute cerebral injury, followed by varying degrees of spontaneous recovery of function, and perhaps a permanent cognitive deficit. In MS, the standard treatment of relapse is a course of corticosteroids or adrenocorticotrophic hormone [1]. However, as of yet there are no medications approved for the treatment of cognitive dysfunction—acute or otherwise—in MS. We might then ask whether a residual deficit ought to be managed as any other cognitive deficit in PwMS. A strong foundation of evidence supports the efficacy of cognitive training and/or rehabilitation in PwMS [1]. Such interventions typically take one of two approaches, namely restorative or compensatory. The former emphasizes specified cognitive domains with repetitive training exercises, whereas the latter focuses on strategy-building to enhance adaptation in real-life situations. Although both approaches are associated with medium-to-large effect sizes when addressing static cognitive dysfunction [60,61], neither has been robustly assessed in the post-acute or early stages of relapse recovery. Such therapy will not temper the severity of cognitive decline during relapse, but some preliminary data suggests a role for cognitive rehabilitation in aiding recovery after relapse. To this end, Giedraitiene et al. demonstrated that improvement on the BVMT-R was significantly greater in cognitively rehabilitated patients as compared to their non-rehabilitated peers [4]. The authors were, however, unable to offer an explanation as to why visual memory was more sensitive to rehabilitation than processing speed or verbal memory. Therefore, future studies should seek to expand upon these findings and determine optimal rehabilitation strategies for each of the various cognitive domains. Moreover, because such interventions are burdensome on both patients and providers, researchers should work to identify factors predicting patients’ responses to therapy in order to facilitate targeted treatment planning.

## 5. Conclusions

In conclusion, the literature contains a substantial body of evidence supporting the existence of cognitive relapse in MS. Taken as a whole, these data suggest that cognition is an independent, often unrecognized component of the MS clinical picture and that objective evidence of cognitive decline indicates active disease, regardless of conventional neurological signs. There is a strong case to be made for the rapid and widespread translation of cognitive screening to clinical routine, as it will empower clinicians to more adequately track disease activity and progression, as well as treatment response. There are, however, glaringly large gaps in our understanding of cognitive relapse. Most notable among these are the lack of knowledge concerning the neuropathology driving cognitive relapse and recovery, and the effect of relapse-adjacent cognitive change on long-term cognitive trajectories. Against this background, additional large-scale prospective studies of cognitive relapse utilizing MRI and evidence-supported cognitive assessment are indicated. 

## Figures and Tables

**Table 1:** The neuropsychological tests and corresponding cognitive domains measured by the Brief Repeatable Battery (BRB) [13], Minimal Assessment of Cognitive Function in Multiple Sclerosis (MACFIMS) [14], and the Brief International Cognitive Assessment for Multiple Sclerosis (BICAMS) [16]. In parenthesis next to each neuropsychological battery is the year in which it was published.

Cognitive Domain	BRB (1990)	MACFIMS (2002)	BICAMS (2012)
Visual processing speed and working memory	SDMT [17]	SDMT [17]	SDMT [17]
Auditory processing speed and working memory	PASAT [25]	PASAT [25]	-
Auditory/verbal memory	SRT [26]	CVLT-II [18]	CVLT-II [18]
Visual/spatial memory	10/36 SPART [13]	BVMT-R [19]	BVMT-R [19]
Language	COWAT [27]	COWAT [27]	-
Spatial processing	-	JLO [28]	-
Executive Function	-	DKEFS–Sorting [29]	-

## Data Availability

No new data were created or analyzed in this study. Data sharing is not applicable to this article.
